# Dissatisfaction with SUS pharmaceutical services and nonadherence to medication use

**DOI:** 10.11606/s1518-8787.2025059006463

**Published:** 2025-12-01

**Authors:** Poliana Vieira da Silva Menolli, Edmarlon Girotto

**Affiliations:** I Universidade Estadual do Oeste do Paraná. Centro de Ciências Médicas e Farmacêuticas. Programa de Pós-Graduação em Ciências Farmacêuticas. Cascavel, PR, Brazil; II Universidade Estadual de Londrina. Centro de Ciências da Saúde. Departamento de Ciências Farmacêuticas. Programa de Pós-Graduação em Saúde Coletiva. Londrina, PR, Brazil

**Keywords:** Patient satisfaction, Pharmaceutical Services, Medication Adherence, Chronic Disease, Primary Health Care

## Abstract

**OBJECTIVE:**

To analyze the association between user dissatisfaction with the pharmaceutical services provided by the Brazilian Unified Health System (SUS) and nonadherence to medication use by patients with chronic non-communicable diseases.

**METHODS:**

A cross-sectional study with data from the service evaluation component of the *Pesquisa Nacional sobre Acesso, Utilização e Promoção do Uso Racional de Medicamentos* in Brazil, conducted from July to December 2014. In-person interviews were analyzed with SUS users aged 18 or over who had used medications for hypertension, diabetes, and dyslipidemia and had visited SUS pharmacies in the three months prior to the survey. The dependent variable was medication nonadherence, assessed through three measures: nonadherence due to lack of medication, nonadherence in the past seven days and declared nonadherence. The independent variable was dissatisfaction with the dimensions of pharmaceutical services (timeliness, availability, and accommodation), measured using item response theory. Logistic regression was performed to calculate crude and adjusted odds ratios (OR).

**RESULTS:**

A total of 2,448 users were evaluated. Nonadherence due to lack of medication was 31.6%; in the past seven days was 11.5%; and declared nonadherence 13.0%. Lack of access and forgetfulness were the main reasons for nonadherence in the past seven days. In the adjusted analysis, dissatisfaction with the “timeliness” dimension was associated with nonadherence in the past seven days (OR = 1.489; 95%CI: 1.023–2.168) and the “accommodation” dimension was associated with declared nonadherence (OR = 3.132; 95%CI: 2.266–4.329).

**CONCLUSION:**

Dissatisfied users were more likely to be nonadherent to medication use across all service dimensions, and dissatisfaction with the availability and accommodation of pharmaceutical services was associated with nonadherence.

## INTRODUCTION

For users of health services, and especially for patients with chronic non-communicable diseases (NCDs), the relationship they have with the services and the professionals in these services is an important factor in building the judgments and beliefs used in decision-making about treatments and the motivation to maintain the recommended health care^1,2^.

Following the therapeutic regimen as prescribed is called “treatment adherence,” which, according to the World Health Organization (WHO), is the extent to which a person’s behavior corresponds with agreed recommendations from a health care provider*,* guidelines ranging from lifestyle changes to the use of medication, and which when fulfilled lead to clinical improvement, reduced mortality and reduced health costs^3^.

Satisfaction is a belief and an expression of attitude towards a service process, which in healthcare is characterized by the patient’s subjective evaluation of their cognitive and emotional reaction resulting from the interaction between expectations, previous experiences, and perception of the actual care received^4,5^.

The evaluation can be positive (satisfaction) or negative (dissatisfaction) and reflects how the patient sees, experiences, and judges the health services received^5^. This perception and judgment involve experience with the services, characteristics of the patients themselves, such as age, their representations of the health-disease process, as well as their understanding of their rights and expectations^1,6^.

Donabedian^7^includes satisfaction as an outcome of health services and even though there is a debate about their ability to assess the quality of services, there is no doubt that it provides valuable information about the health experience from the patient’s perspective^4^. Users who are satisfied with health services tend to have a better quality of life, provide important information to the health provider, return to the service when necessary, and are more adherent to the prescribed treatments^4,8^.

Medical consultations and procedures, hospital admissions and nursing care are the services most frequently observed in user satisfaction evaluations^1^, but with the organization of services in health care networks and the focus on multiprofessional care for NCD patients, there is an urgent need to know and evaluate other professionals and services in the patient’s itinerary^9^.

Pharmaceutical care is part of Brazilians’ comprehensive right to health and its aim is to provide qualified, equal, and universal access to medicines and to promote their rational use. It is considered a strategic action within health care in the Brazilian Unified Health System (SUS) and today it is organized beyond its initial product-focused cycle, with the construction of pharmaceutical services (PS) that seek to be patient-centered^10^. PS may be technical-managerial, involving the management of products and services that support healthcare networks, or technical-care, involving direct care for patients and communities aimed at improving clinical outcomes, promoting treatment adherence, and preventing adverse events^10^. Accessible, quality pharmaceutical services promote user satisfaction^11,12^.

Medication adherence is not easy to measure, there is no gold standard to compare it to, and the large number of methods and interpretations of the measures make it difficult to reach a consensus^13^. Adherence/nonadherence is a complex and dynamic event, it varies over time according to the type of disease and the number of medications^14^, which is why the assessment of the dosing history of each medication is currently believed to be more accurate^15^. Measurements made at a static point in time and by self-report, although criticized, are still the only method available to all health professionals and are able to signal and evaluate important components of adherence^14^.

In Brazil, there are few studies evaluating the relationship between user satisfaction with health services and medication nonadherence^16^, and to date, there are none that evaluate dissatisfaction with PS and its influence on nonadherence exclusively. In this context, the aim of this study was to analyze the association between user dissatisfaction with SUS PS and medication nonadherence by patients with NCDs who used medications for hypertension, diabetes, and dyslipidemia.

## METHODS

This is a cross-sectional study, with data from the services component of *Pesquisa Nacional sobre Acesso, Utilização e Promoção do Uso Racional de Medicamentos* (PNAUM – National Survey on Access, Use, and Promotion of Rational Use of Medicines), which characterized the organization of pharmaceutical services in SUS primary health care (PHC) in relation to access and promotion of the rational use of medicines, and to identify and discuss the factors that interfere with the consolidation of pharmaceutical services at the municipal level. The data was collected from July to December 2014 and details of the PNAUM Services methodology, as well as the sampling process, are described in Álvares et al.^19^

PNAUM Services interviewed 8,803 SUS users over the age of 17, who were in PHC services waiting for a medical appointment, were able to answer the proposed questions and agreed to take part in the survey.

This study followed the guidelines of The Strengthening the Reporting of Observational Studies in Epidemiology (STROBE)^20^and analyzed in-person interviews conducted with SUS PHC users aged 18 or over who reported using medications for the treatment of systemic arterial hypertension (SAH), diabetes, and dyslipidemia and who had used SUS pharmacies in the three months prior to the survey. This sample was chosen because they are long course treatments, already initiated previously, which make the patient an experienced actor in contact with the PS and for which nonadherence to treatment is a major problem for patients and health services^3^.

The therapeutic groups of the WHO Anatomical Therapeutic Chemical Classification were considered as treatment: for diabetes the drugs belonging to therapeutic group A10 (drugs used in diabetes); for SAH the drugs in therapeutic groups C2 (antihypertensives), C3 (diuretics), C4 (peripheral vasodilators), C7 (beta-blocking agents), C8 (calcium channel blockers), C9 (agents acting on the renin-angiotensin system); and for dyslipidemia the therapeutic group C10 (lipid-modifying agents)^21^.

The instrument’s questions on user satisfaction were based on the Long-Form Patient Satisfaction Questionnaire (PSQ-III), adapted for pharmaceutical services, according to Soeiro et al.^12^

All of the PNAUM databases are public, but they are no longer publicly available, and access must be requested from the Ministry of Health. The service evaluation database for this study was obtained in 2020.

### Variables

The interviewees were characterized according to: region of Brazil (North, Northeast, Midwest, South, and Southeast), sex (female and male), race/skin color (White/Yellow and Black/Brown/Indigenous), marital status (with a partner and without a partner), schooling (0 to 8 years and over 8 years of schooling), age (17 to 39, 40 to 59 and equal to or over 60), number of self-reported illnesses (1 to 5 and over 5), polypharmacy – use of four or more medicines (yes and no), the need for assistance to take medicines (yes and no), emergency care in the past 12 months (yes and no), and hospitalization in the past 12 months (yes and no).

### Assessment of Medication Nonadherence

Nonadherence to treatment was assessed on a one-off basis using three variables that signaled the behavior of not using the prescribed medication: nonadherence due to lack of medication (NAL), nonadherence in the past seven days (NA7), and declared nonadherence (DNA).

The NAL variable assessed the difficulty in obtaining the medication, indirectly pointing to the non-use of medication, and was constructed from the question: “*Did you have any problems obtaining this medication*?” which allowed for “yes” and “no” answers. NA7 assessed non-use of the medication in the previous week and was based on the question: “*Have you stopped taking this medication for any reason in the past 7 days*?” which allowed for “yes” and “no” answers. The DNA variable assessed declared and/or intentional non-adherence, when the patient declares that they have stopped taking the medication of their own accord, through the question: “*Do you stop using any medication prescribed by your doctor?”* with “yes” and “no” answers.

The NAL and NA7 variables were constructed from questions for each medicine used by the interviewee. In the questions for each medicine, the “yes” answers (problem in getting and stopping taking them) were given a value of 1 and the “no” answers were given a value of 0. The values were added up and divided by the total number of medicines used by the patient. For the NAL and NA7 variables, a result greater than 0.2 was considered nonadherence (more than 20% of medicines with problems in being obtained or not taken), a value based on the adherence concept of Sackett et al.^22^and WHO^3^. The NA7 also looked at the reasons given by the patient for having stopped taking the medication the previous week. The DNA variable was constructed using a single question per interviewee, with “yes” answers considered nonadherence.

The variables were assessed in isolation, meaning that the same patient could be nonadherent in all three measures.

### Assessment of User Dissatisfaction

User dissatisfaction with the PS was assessed using the theoretical model developed by Esher^11^to assess user satisfaction with the dispensing of HIV medication, adapted for the assessment of the PS^12^.

In this model, satisfaction is mediated by access (the relationship between obstacles encountered by the user in the search for health care and the population’s ability to overcome these obstacles), so more access is considered satisfaction and less is considered dissatisfaction, which makes dissatisfaction the direct opposite of satisfaction. Dispensing and pharmaceutical services can be assessed operationally through access, since problems in the dimensions of access to health services and products influence users and the system itself, modifying user satisfaction and changing the patterns of organization and use of services^11^.

As dispensing is considered a key stage of the PS, in which all the previous stages are effectively put into the patients’ health care routine and because it is the stage that is the link for monitoring the use of medicines, this research used the user satisfaction assessment model created by Esher^11^to assess dissatisfaction with the PS in SUS PHC.

In the analysis, the following dimensions of access were used: timeliness/convenience (TI/CO), availability (AVAI) and accommodation (ACCO), which are the criteria used by the user to judge satisfaction, focusing on the current experience with the service and not on its effect on health. The ACCO dimension was divided into the sub-dimensions technical quality of dispensing, technical quality of the medication, ambience and interpersonal aspects^11,12^.

The response variables were grouped into the three dimensions: TI/CO, AVAI and ACCO, and dichotomized, considering “0” as satisfied and “1” as dissatisfied (Box).

**Box:** Construct of variables related to user dissatisfaction with pharmaceutical services.

Dimension	Subdimension	Variable	Questions
**Timeliness** Existence of services in the place and at the time when it is technically required by the users’ health situation.	**Convenience** Distance/ease of geographic access, organization of the service, time spent by the user and the possibility of choosing health units	Distance from home	*Is this place far from your home?* (Assumes a value of 0 for the answer “no” and 1 for the answers “yes” and “more or less”).
		Difficulty getting to the service	*Getting here is* (“Very easy” and “Easy” were coded 0, the answers “Neither easy nor difficult,” “Difficult,” and “Very difficult” were coded 1).
		Opening hours	*The opening hours of this Health Unit are*: (“Very good” and “Good” were coded 0; the answers “Neither bad nor good,” “Bad,” and “Very bad” were coded 1*)*.
		Waiting times	*How long do you usually wait to get your medication from SUS public pharmacies?* (The answers “Don’t wait” and “A little” were coded 0 and the answer “A long time” was coded 1*)*.
**Availability** Relationship between the type and quantity of products and services needed and the type and quantity of services offered	**Presence of medicine** Availability of the medicine in the pharmacy in the quantity prescribed for the user	Got free medicine	*Where did you get this medicine last time?* (Code 0 for the answers “SUS pharmacy,” “Popular pharmacy,” or “Church or union” (which would be related to facilitating access to the medicine, promoting satisfaction) and 1 for the answer “Commercial pharmacy”). In the case of patients who took more than one medicine, if there was any medicine they stopped taking, their answer would be 1*)*.
		Problems getting medicine	*Did you have any problems getting medicine last time?* (Code 0 for the answer “no problem” and 1 for answers indicating that the patient had problems getting the medicine). In the case of patients who took more than one medicine, if there was a medicine they stopped taking, their answer would be 1).
		Stopped taking in the past seven days	*Have you stopped taking the medicine for any reason in the past seven days?* (Assumes 0 for no and 1 for yes). In the case of patients who took more than one medicine, if there was a medicine they stopped taking, their answer would be 1).
		Medicines in SUS in the past three months	*In the past three months, how often did you get the medicines you were looking for from SUS public pharmacies?* (The answers “always” and “repeatedly” were coded 0; the answers “sometimes,” “rarely,” and “never” were coded 1).
		Difficulties with the medicine from 1 to 6	*1. Did you have difficulty remembering to take your medication? 2. Did you have difficulty managing your medication because you take many pills a day? 3. Did you have difficulty managing your medication because it is difficult to obtain? 4. Did you have difficulty managing your medication because it is difficult to read the information on the packaging? 5. Did you have difficulty managing your medications because it is difficult to fit their use into your work schedule? 6. Did you have difficulty managing your medications because there are different medications with the same shape and color? The answer “no” received code 0 and the answer “yes” received code 1.*
**Accommodation** Fit between the characteristics of products and services and users’ expectations and needs	**Technical quality of dispensing** Competence of providers and adherence to the essential components of dispensing.	Receive information	*When you pick up medicines from SUS public pharmacies, do the staff who deliver the medicines pass on information about how to use them?* The answer “yes” was coded 0 and the answers “sometimes” and “no” were coded 1.
		Understand information	*Do you understand the information passed on by the staff who deliver medicines to SUS public pharmacies?* The answers “always” and “repeatedly” were coded 0 *and* the answers “sometimes,” “rarely,” and “never” were coded 1.
		Pharmacist/ employee available	*Is the pharmacist or another SUS public pharmacy employee available when you need to ask questions about medicines?* The answers “always” and “repeatedly” were coded 0 *and* the answers “sometimes,” “rarely,” and “never” were coded 1.
		Guidance on storage at home	*When you pick up medicines from SUS public pharmacies, do you receive guidance on how to store them at home?* The answers “always” and “repeatedly” were coded 0, the answers “sometimes,” “rarely,” and “never” were coded 1.
	**Technical quality of the medicine** Characteristics of the packaging and presentation of the medicines, the production of adverse reactions and the possible resolution of symptoms.	SUS *versus* commercial effect	*For you, the effects of medicines received in public SUS pharmacies compared to the effects of medicines bought in commercial pharmacies are*: The answers “the same” and “better” received code 0 and the answer “worse” code 1.
		Effect of the medicine	*How is this medicine working for your illness?* Code 0 for the answer “well” and 1 for the answers “fairly well” and “not well.” In the case of patients who took more than one medicine, if there was a medicine that they thought worked fairly well or not well, their answer would be 1.
		Causes health problem	*In your opinion, does this medicine cause any health problems for you?* Code 0 for “no” and 1 for “yes.” In the case of patients who took more than one drug, if there was a drug that caused a problem, their answer would be 1.
	**Ambience** Characteristics of the place where care is provided and must ensure that the facilities meet the minimum conditions for a comfortable environment (cleanliness, signage, sufficient ventilation, a place protected from the weather, clean drinking water).	Signage	*How do you rate the signposting to find the SUS public pharmacy?* The answers “very easy” and “easy” were coded 0 *and* the answers “neither easy nor difficult,” “difficult,” and “very difficult” were coded 1.
		Cleanliness	*How do you rate the cleanliness of the SUS public pharmacy?* The answers “very good” and “good” were coded 0 and the answers “neither bad nor good,” “bad,” and “very bad” were coded 1.
		Comfort	*How do you rate the comfort of the SUS public pharmacy?* The answers “very good” and “good” were coded 0 *and* the answers “neither bad nor good,” “bad,” and “very bad” were coded 1.
		Service	*How do you rate the service provided by the SUS public pharmacy?* The answers “very good” and “good” were coded 0 and the answers “neither bad nor good,” “bad,” and “very bad” were coded 1.
	**Interpersonal relations** Dispensers’ attitude towards users when supplying medicines and includes the components of autonomy, dignity, and confidentiality.	Respect/courtesy	*Do SUS public pharmacy staff treat you with respect and courtesy?* The answers “always” and “repeatedly” were coded 0 and the answers “sometimes,” “rarely,” and “never” were coded 1.
		Privacy	*Do you feel that the service provided in the SUS public pharmacy ensures privacy?* The answers “always” and “repeatedly” were coded 0 and the answers “sometimes,” “rarely,” and “never” were coded 1.
		Service evaluation	*How do you rate the service provided by the SUS public pharmacy?* The answers “very good” and “good” were coded 0 and the answers “neither bad nor good,” “bad,” and “very bad” were coded 1.

SUS:Brazilian Unified Health System.

The variables in the TI/CO and ACCO dimensions were constructed from a single question per interviewee. In the AVAI dimension, the variables were constructed using questions per medication used, so the values for dissatisfaction (1) in each question were added up and divided by the number of medications. Results equal to or above 0.5 were considered dissatisfied and classified as “1” and values below 0.5 satisfied and classified as “0”.

In order for user dissatisfaction to be represented by a latent variable of each dimension, originally not observed, the item response theory (IRT) technique was used^23^. The dissatisfaction index for each dimension was created and transformed into a scale from 0 to 1, where proximity to 0 indicated greater satisfaction and to 1, greater dissatisfaction.

The median was used to dichotomize the answers for each of the variables, with higher or equal values being coded 1 (dissatisfied) and lower values 0 (satisfied). The IRT models were built in the R program version 4.1.2, using the *lt* package^23^. The significance level adopted was 5%.

### Data Analysis

The data was processed in Microsoft Excel, and was exported and analyzed using IBM Statistical Package for the Social Sciences*®* (IBM-SPSS) version 19.0, using the CSPLAN commands, appropriate for complex samples^19^, which takes into account sample weights and design effects. Descriptive analysis was performed by presenting relative frequencies and confidence intervals of relative frequencies. The association between the adjustment variables, chosen for their influence on adherence^24^, and the three outcomes was measured using Pearson’s *X* ^2^test. Finally, after ruling out the interaction effect of the independent variables with the adjustment variables on treatment adherence measures, regression analyses were carried out.

To analyze the relationship between user dissatisfaction and nonadherence to treatment, crude and adjusted logistic regression was performed and the results were presented using OR. The model was adjusted using the variables that obtained a result of p < 0.20 in the analysis of association (Pearson’s *X* ^2^test), independently for each outcome tested. The gender variable was included in all the adjustment models regardless of the p-value in the association analysis. The level of statistical significance set in this study was 5% (p < 0.05).

The PNAUM was approved by the National Research Ethics Committee (CONEP) under Opinion No. 398.131, dated September 16, 2013. The interviews were carried out after the objectives of the study had been explained and the interviewee or legal guardian had agreed to them by signing an informed consent form.

## RESULTS

Of the 8,803 people interviewed by PNAUM Services, we excluded 2,303 who did not use medication, 3,567 who used medication that was not treatment for diabetes, hypertension and dyslipidemia, 450 interviewees who had not used SUS pharmacies in the three months prior to the survey and 35 who answered “I don’t know” to the questions about medication adherence (outcome). Thus, 2,448 users were assessed.

The majority were female (73.9%); over 60 years old (47.5%); Black, Brown or Indigenous (57.0%); with a partner (67.8%); with up to eight years of schooling (80.5%); reported having up to five diseases (88.3%); using one to three medicines (68.4%) and did not need help to take their medication (90.1%); and lived in the Southeast (38.5%) and South (26.1%) regions (Table 1).

**Table 1:** Characterization of patients undergoing treatment for systemic arterial hypertension, diabetes and dyslipidemia treated by the pharmaceutical services of the PNAUM service evaluation component, 2014, (n = 2,448 people).

Characterization and adjustment variables	Total (%)	Nonadherence due to lack of access		Nonadherence 7 days		Declared nonadherence	
		%	p^a^	%	p^a^	%	p^a^
Region of Brazil			0.110		0.643		0.214
North	3.6	41.3		13.0		10.8	
Northeast	27.3	35.4		10.4		14.9	
Midwest	4.3	34.0		14.7		12.9	
South	26.1	26.3		12.3		14.2	
Southeast	38.5	31.2		11.3		11.0	
Sex			0.680		0.022		0.451
Female	73.9	32.9		12.6		12.6	
Male	26.1	27.7		8.4		14.1	
Race/skin color			0.509		0.918		0.266
White/Yellow	43.0	32.4		11.5		14.1	
Black/Brown/Indigenous	57.0	30.7		11.6		12.0	
Marital status			0.328		0.601		0.718
With a partner	67.8	32.4		11.8		13.2	
Without a partner	32.2	29.8		10.9		12.5	
Schooling			0.003		< 0.001		0.008
0 to 8 years	80.5	29.8		10.0		14.1	
Over 8 years old	19.5	38.9		17.7		8.3	
Age (in years)			0.544		< 0.001		0.145
17 a 39	9.3	29.0		18.9		18.3	
40 a 59	45.0	33.0		14.6		11.8	
≥ 60 years	45.7	30.7		7.0		13.0	
Number of illnesses			0.443		0.996		0.571
0 or don’t know	8.2	34.4		11.4		10.6	
1 a 5	88.3	31.0		11.5		13.3	
Above 5	3.5	38.0		11.9		10.5	
Polypharmacy			< 0.001		0.274		0.353
Yes	31.6	39.8		12.9		11.8	
No	68.4	27.8		10.9		13.5	
Need help taking medication			0.151		0.181		0.739
Yes	9.9	37.1		8.3		13.9	
No	90.1	31.0		11.9		12.9	
Emergency care (past 12 months)			0.003		< 0.001		0.020
Yes	23.5	38.0		18.4		9.4	
No	76.5	29.6		9.4		14.1	
Hospitalization (past 12 months)			0.090		0.010		0.054
Yes	11.5	37.3		17.6		8.7	
No	88.5	30.8		10.7		13.5	

PNAUM: *Pesquisa Nacional sobre Acesso, Utilização e Promoção do Uso Racional de Medicamentos* (National Survey on Access, Use, and Promotion of Rational Use of Medicines).

^a^ Pearson’s *X* ^2^ test.

NAL was identified in 31.6%, NA7 in 11.5% and DNA in 13.0% of the interviewees. The justifications for not using the medication in the previous week were not having the medication (no prescription, no appointment, shortage at the basic health unit, shortage at the pharmacy and no money to buy it) (50.2%), followed by forgetfulness (22.9%), presence of unwanted effects (9.3%), own initiative (7.3%), among others.

NAL was higher among users with a higher level of education, who reported polypharmacy, and who had received emergency care or reported being hospitalized in the past 12 months. NA7 was more prevalent in women, in users with higher levels of education and younger age, and in those who had received emergency care or reported hospitalization in the past 12 months. DNA, on the other hand, was higher among users with lower levels of education and those who had not received emergency care or reported being hospitalized in the past 12 months (Table 1).

Most users were satisfied with the PHC pharmaceutical services in the municipalities and the highest rates of dissatisfaction were found in the variables *difficulty in reading the packaging*, in the availability dimension (AVAI), with 41.8% of dissatisfied users, followed by *distance from home*, in the Timeliness/Convenience dimension (TI/CO), with 38.6% of dissatisfied users and *being able to obtain medicines from the SUS*, with 36.5% of dissatisfied users (Table 2).

**Table 2:** Prevalence of nonadherence to medication use according to the dissatisfaction variables of users served by PHC PS. PNAUM. 2014. (n = 2,448).

Dimension/subdimension of dissatisfaction	Prevalence % (95%CI)			
	Total %	Nonadherence due to lack	Nonadherence 7 days	Declared nonadherence
Timeliness/convenience				
Distance from home				
Satisfied	61.4 (58.8–63.9)	31.5 (28.5–34.7)	10.2 (8.5–12.3)	12.9 (10.8–15.3)
Dissatisfied	38.6 (36.1–41.2)	31.6 (27.7–35.7)	13.4 (10.9–16.8)	13.1 (10.4–16.3)
Difficulty getting to the service				
Satisfied	82.4 (80.2–84.3)	30.7 (28.1–33.4)	10.2 (8.7–12.0)	12.9 (11.1–15.0)
Dissatisfied	17.6 (15.7–19.8)	35.5 (29.7–41.8)	17.5 (13.2–22.9)	13.4 (9.6–18.4)
Opening hours				
Satisfied	89.7 (88.0–91.2)	30.2 (27.7–32.8)	10.8 (9.2–12.6)	12.8 (11.0–14.8)
Dissatisfied	10.3 (8.8–12.0)	43.2 (35.3–51.4)	18.3 (12.8–25.4)	15.1 (10.1–22.0)
Waiting times				
Satisfied	94.8 (93.5–95.9)	30.9 (28.5–33.5)	11.4 (9.8–13.2)	13.2 (11.5–15.2)
Dissatisfied	5.2 (4.1–6.5)	43.8 (32.7–55.5)	12.8 (6.9–22.5)	9.4 (4.7–17.8)
Availability/presence of the medicine				
Got the medication from SUS				
Satisfied	63.5 (61.0–65.9)	13.1 (11.0–15.5)	7.5 (6.0–9.4)	14.3 (12.1–16.8)
Not satisfied	36.5 (34.1–39.0)	63.7 (59.5–67.6)	18.5 (15.4–22.1)	10.7 (8.3–13.6)
Problems getting the medication				
Satisfied	64.5 (62.0–67.0)	-	9.0 (7.4–11.0)	13.5 (11.4–16.0)
Unsatisfied	35.5 (33.0–38.0)	89.0 (86.1–91.4)	16.1 (13.1–19.5)	12.0 (9.4–15.1)
Stopped taking it in the past seven days				
Satisfied	85.5 (83.5–87.3)	31.9 (29.3–34.6)	10.9 (9.3–12.8)	9.7 (8.1–11.5)
Dissatisfied	14.5 (12.7–16.5)	29.7 (23.7–36.5)	15.0 (10.7–20.6)	32.1 (26.0–39.0)
Medicines in the SUS in the past three months				
Satisfied	73.8 (71.5–76.1)	33.4 (30.6–36.4)	10.6 (8.9–12.6)	11.2 (9.4–13.3)
Dissatisfied	26.2 (23.9–28.5)	23.6 (22.1–31.0)	14.0 (10.9–18.0)	17.9 (14.3–22.1)
Availability/presence of the medicine/difficulties with the medicine				
Did you have trouble remembering to take the medicine?				
Satisfied	71.2 (68.8–73.5)	31.4 (28.6–34.4)	11.5 (9.7–13.6)	13.3 (11.3–15.6)
Dissatisfied	28.8 (26.5–31.2)	31.9 (27.6–36.5)	11.6 (8.9–14.9)	12.1 (9.3–15.5)
Did you find it difficult to handle medication because you use a lot of pills a day?				
Satisfied	77.7 (75.5–79.8)	30.8 (28.1–33.7)	11.5 (9.7–13.5)	14.3 (12.3–16.6)
Not satisfied	22.3 (20.2–24.5)	34.4 (29.5–39.7)	10.8 (7.9–14.5)	8.4 (6.0–11.6)
Have you found it difficult to deal with medication because it’s hard to get hold of?				
Satisfied	73.4 (71.1–75.6)	25.5 (22.9–28.3)	10.2 (8.5–12.1)	14.0 (11.9–16.3)
Dissatisfied	26.6 (24.4–28.9)	48.3 (43.4–53.2)	15.3 (12.0–19.2)	10.2 (7.6–13.4)
Did you find it difficult to deal with medicines because it’s hard to read what’s written on the packaging?				
Satisfied	58.2 (55.6–60.8)	32.3 (29.2–35.6)	10.1 (8.3–12.3)	12.4 (10.2–14.8)
Dissatisfied	41.8 (39.2–44.4)	30.5 (26.8–34.4)	13.5 (11.0–16.5)	13.9 (11.3–17.0)
Did you find it difficult to deal with medicines because it is difficult to match their use with your work?				
Satisfied	85.9 (84.1–87.6)	31.1 (28.6–33.8)	11.5 (9.8–13.4)	13.4 (11.6–15.5)
Dissatisfied	14.1 (12.4–15.9)	32.4 (26.4–38.9)	11.9 (8.4–16.6)	10.7 (7.3–15.4)
Did you find it difficult to deal with medicines because there are different medicines with the same shape and coloring?				
Satisfied	81.5 (79.5–83.4)	30.8 (28.1–33.5)	12.0 (10.2–14.0)	13.2 (11.3–15.4)
Dissatisfied	18.5 (16.6–20.5)	35.5 (30.0–41.3)	9.3 (6.6–13.0)	12.3 (8.9–16.7)
Accommodation/technical quality of dispensing				
You receive information				
Satisfied	72.8 (70.4–75.1)	32.0 (29.2–35.0)	11.1 (9.4–13.2)	9.7 (8.0–11.7)
Dissatisfied	27.2 (24.9–29.6)	30.7 (26.3–35.5)	12.7 (9.8–16.3)	21.6 (17.7–26.1)
Understands the information				
Satisfied	94.9 (93.5–96.0)	31.4 (28.7–34.3)	11.2 (9.4–13.2)	10.3 (8.5–12.3)
Unsatisfied	5.1 (4.0–6.5)	35.7 (24.6–48.5)	15.4 (8.4–26.6)	13.5 (6.9–24.8)
Pharmacist/employee available				
Satisfied	82.5 (80.4–84.5)	31.9 (29.2–34.8)	11.0 (9.4–13.0)	10.2 (8.5–12.2)
Not satisfied	17.5 (15.5–19.6)	31.4 (25.8–37.7)	12.4 (8.8–17.2)	19.8 (15.1–25.5)
Guidance on how to store at home				
Satisfied	74.8 (72.2–77.2)	32.2 (29.0–35.4)	11.2 (9.2–13.5)	8.6 (6.8–10.7)
Unsatisfied	25.2 (22.8–27.8)	30.2 (25.2–35.8)	12.3 (9.1–16.4)	16.9 (13.0–21.6)
Accommodation/technical quality of the medicine				
SUS *versus* commercial effect				
Satisfied	92.8 (91.1–94.1)	30.6 (28.0–33.4)	11.6 (9.9–13.5)	11.0 (9.3–13.0)
Dissatisfied	7.2 (5.9–8.9)	31.0 (22.0–41.7)	16.7 (10.0–26.6)	29.0 (20.3–39.6)
Medication effect				
Satisfied	87.5 (85.7–89.2)	31.3 (28.7–33.9)	10.5 (9.0–12.3)	12.8 (11.0–14.8)
Dissatisfied	12.5 (10.8–14.3)	33.6 (27.0–41.0)	18.6 (13.6–24.9)	14.1 (9.6–20.4)
Causes health problems				
Satisfied	92.7 (91.2–93.9)	31.2 (28.7–33.8)	11.2 (9.6–13.0)	12.6 (10.9–14.5)
Dissatisfied	7.3 (6.1–8.8)	36.4 (27.9–45.8)	15.5 (10.2–22.9)	17.7 (11.5–23.6)
Accommodation/ambience				
Signage				
Satisfied	91.2 (89.6–92.6)	31.7 (29.2–34.3)	11.6 (10.0–13.4)	11.4 (9.8–13.3)
Dissatisfied	8.8 (6.6–9.5)	30.3 (22.7–39.1	12.0 (7.2–19.5)	29.2 (21.7–38.1)
Cleanliness				
Satisfied	92.0 (90.5–93.4)	31.5 (29.0–34.1)	11.1 (9.5–12.9)	11.0 (9.4–12.9)
Dissatisfied	8.0 (6.6–9.5)	30.6 (22.5–40.0)	14.2 (8.7–22.4)	26.1 (18.5–35.5)
Comfort				
Satisfied	74.6 (72.2–76.8)	31.5 (28.7–34.4)	11.2 (9.5–13.2)	9.9 (8.2–11.9)
Dissatisfied	25.4 (23.2–27.8)	32.5 (27.8–37.5)	12.9 (9.8–16.9)	21.7 (17.7–26.4)
Service				
Satisfied	91.3 (89.7–92.7)	31.5 (29.0–34.1)	11.4 (9.8–13.2)	11.3 (9.7–13.2)
Dissatisfied	8.7 (7.3–10.3	31.9 (24.1–40.8)	13.7 (8.6–21.1)	30.9 (23.2–39.8)
Accommodation/interpersonal relations				
Respect/courtesy				
Satisfied	93.8 (92.4–94.9)	31.5 (29.1–39.1)	11.4 (9.8–13.2)	11.5 (9.8–13.3)
Dissatisfied	6.2 (5.1–7.6)	33.1 (23.9–43.8)	13.9 (8.0–22.9)	35.6 (26.3–46.2)
Privacy				
Satisfied	71.4 (68.9–73.8)	32.1 (29.2–35.1)	10.3 (8.6–12.4)	9.6 (7.9–11.7)
Dissatisfied	28.6 (26.2–31.1)	30.9 (26.4–35.7)	13.9 (10.8–17.8)	20.2 (16.4–24.5)
Service evaluation				
Satisfied	91.3 (89.7–92.7	31.5 (29.0–34.1)	11.4 (9.8–13.2)	11.3 (9.7–13.2)
Dissatisfied	8.7 (7.3–10.3)	31.9 (24.1–40.8)	13.7 (8.6–21.1)	30.9 (23.2–39.8)

95%CI: 95% confidence interval; SUS: Unified Health System; PHC: primary health care; PNAUM: *Pesquisa Nacional sobre Acesso, Utilização e Promoção do Uso Racional de Medicamentos* (National Survey on Access, Use, and Promotion of Rational Use of Medicines); PS: pharmaceutical services.

NAL was higher across the dimensions studied, both among the satisfied and the dissatisfied users, with AVAI showing the highest rates. NA7 was higher among the dissatisfied users in all dimensions. The DNA was also higher among the dissatisfied users, especially in the Accommodation (ACCO) dimension, whose rates were three times higher than those observed for satisfied users (Table 2).

The TI/CO dimension was associated with NA7 in the crude and adjusted analysis (OR = 1.489; 95%CI: 1.023–2.168; p = 0.038). The ACCO was associated with DNA in the crude analysis, which was confirmed in the adjusted analysis (OR = 3.132; 95%CI: 2.266–4.329; p < 0.001). Table 3 shows the results of the logistic regression between the dimensions of satisfaction and adherence.

**Table 3:** Crude and adjusted logistic regression of the dimensions of dissatisfaction with PHC pharmaceutical services and nonadherence to the use of medication for hypertension, diabetes, and dyslipidemia. Brazil 2014.

Nonadherence lack of access					
Dimensions	% (95%CI)	OR (95%CI)	p	OR_a_ (95%CI)^a^	p
Timeliness					
Satisfied	29.9 (26.7–33.3)	1.000		1.000	
Dissatisfied	33.4 (29.9–37.0)	1.313 (0.994–1.734)	0.055	1.260 (0.947–1.678)	0.113
Availability					
Satisfied	24.5 (21.5–27.8)	1.000		1.000	
Unsatisfied	39.5 (35.9–43.2)	1.294 (1.000–1.674)	0.050	1.188 (0.911–1.549)	0.204
Accommodation					
Satisfied	31.3 (28.0–34.8)	1.000		1.000	
Unsatisfied	31.9 (28.4–35.5)	0.914 (0.706–1.184)	0.496	0.899 (0.689–1.173)	0.431
**Nonadherence 7 days**					
**Dimensions**	**% (95%CI)**	**OR (95%CI)**	**p**	**OR_a_ (95%CI)^b^**	**p**
Timeliness					
Satisfied	9.6 (7.7–11.8)	1.000		1.000	
Dissatisfied	13.6 (11.2–16.4)	1.673 (1.152–2.428)	0.007	1.489 (1.023–2.168)	0.038
Availability					
Satisfied	9.2 (7.3–11.4)	1.000		1.000	
Not satisfied	14.2 911.8–17.0)	0.963 (0.665–1.394)	0.841	0.923 (0.628–1.357)	0.684
Accommodation					
Satisfied	10.1 (8.2–12.5)	1.000		1.000	
Unsatisfied	13.1 (10.8–15.8)	1.142 (0.799–1.632)	0.466	1.204 (0.839–1.729)	0.314

**Dimensions**	**% (95%CI)**				**p**
Timeliness					
Satisfied	12.8 (10.6–15.5)	1.000		1.000	
Dissatisfied	13.1 (10.7–15.9)	1.102 (0.745–1.630)	0.627	1.117 (0.751–1.661)	0.585
Availability					
Satisfied	14.6 (12.1–17.4)	1.000		1.000	
Not satisfied	11.2 (9.1–13.8)	0.789 (0.543–1.145)	0.212	0.815 (0.558–1.189)	0.288
Accommodation					
Satisfied	7.0 (5.3–9.1)	1.000		1.000	
Unsatisfied	19.6 (16.7–22.8)	3.081 (2.228–4.258)	< 0.001	3.132 (2.266–4.329)	< 0.001

PHC: primary health care; OR: odds ratio; ORa: adjusted odds ratio; 95%CI: 95% confidence interval.

^a^ Adjusted for region, gender, schooling, polypharmacy, help, emergency, and hospitalization.

^b^ Adjusted for sex, schooling, age, help, emergency, and hospitalization.

^c^ Adjusted for gender, schooling, age, emergency, and hospitalization.

## DISCUSSION

The results showed that nonadherence, whether due to access difficulties the last time, non-use in the previous week or declared nonadherence, is a more prevalent event among those dissatisfied with the services in all the dimensions studied and is associated with dissatisfaction with the timeliness and accommodation dimensions.

Dissatisfaction with the AVAI dimension, which assessed the barriers faced by users in obtaining the medication from the health service and using it at home, was not associated with nonadherence. However, as we look at the NAL values, which are three times higher than the other measures (NA7 and DNA), the lack of medication appearing as the main justification for NA7 and the prevalence of nonadherence in the AVAI dimension being higher among dissatisfied users, we can see the difficulty of accessing medications in PHC in Brazil, a determining factor for non-use of medications and user dissatisfaction^25,26^.

Free access via the SUS serves a significant portion of the population and has improved over the years, but difficulties remain^25^. The difficulty in obtaining medications regularly and free of charge has been associated with nonadherence in other studies in Brazil^25^.

The 2019 National Health Survey showed that 85% of Brazilians are able to obtain all the medicines they need, through public and/or private channels. The proportion of people who obtained at least one prescription drug through the public sector alone was 30.5%, demonstrating that there are obstacles to public access^30^. The difficulty of public access is confirmed by the results of the SHA Accounts assessment for Brazil (health accounts from the perspective of international accounting), which showed that, from 2015 to 2019, around 90% of spending on medicines and medical items came directly from families’ pockets^31^.

Dissatisfaction with the TI/CO dimension, associated with NA7 and forgetfulness, was the main reason given by the interviewees, excluding the lack of medication. This association, i.e., dissatisfaction with the existence of services where and when the user needs them and forgetfulness, may be related to the construction of negative beliefs about health services, including the experiences of searching for medicines in dispensing services^32,33^. Concerns about treatment are often “masked” behind “forgetfulness.” More in-depth studies show that negative beliefs about medicines and health services work to promote forgetfulness^32,33^.

Another important factor is that forgetfulness, characterized as unintentional nonadherence, seems not to be so unconscious or random. Forgetfulness can function as a first micro-experiment for future intentional nonadherence, when the patient actively decides to renounce the prescribed therapy^2,34^.

Dissatisfaction with the ACCO dimension was associated with DNA, with nonadherence among dissatisfied users being three times that of satisfied ones. This can be explained by the fact that patients who openly declare that they don’t follow their medication prescriptions are characterized as intentional non-adherents, who are significantly more likely to doubt their personal need for the medication, have greater concerns about taking it, as well as being more critical of the differences between expectations and the reality of the services^2,34^. A possible inverse relationship between DNA and ACCO cannot be ruled out with cross-sectional data, since the factor and the outcome were collected at the same time, but other studies place dissatisfaction as a predictor of nonadherence^35,36^.

A study by Barreto et al.^17^with patients treated at 90 PHC services (basic health units and family health units) and taking antihypertensive drugs found an association between dissatisfaction with PHC services and nonadherence to hypertension treatment. The services that caused the most dissatisfaction were the reception service and the scheduling of medical appointments. In Syria^37^, nonadherence to antihypertensive medication was associated with dissatisfaction with health services, consultation time, the doctor’s explanation of the disease and the lack of (free) medication in dispensing units. Lihara et al.^34^found an association between dissatisfaction with the healthcare team, especially interpersonal relationships, and nonadherence to the use of medication for CNCDs in Japan.

In the literature, the influence of satisfaction with health services on medication adherence is explained by the models of self-regulation in health^35^and health beliefs, which aim to explain and predict health behaviors, and which are similar to each other^38^. The health beliefs model does this through six dimensions of human perception: perceived susceptibility, perceived severity, perceived benefits, perceived barriers, self-regulation, and stimuli for action. In these, factors such as beliefs, experience with health services, related to the disease, treatments and patients influence both satisfaction and adherence^38^.

Adherence is a more complex health behavior than judging oneself satisfied and involves direct and mediating predictors, and satisfaction has been observed as both. What is known is that satisfaction is a component of adherence, the more dissatisfied the less adherent, but it is still unclear whether satisfaction is a casual characteristic of adherents^35,38^.

In this study, dissatisfaction was constructed by evaluating access to products and services by users of PHC medicines. The accumulation of barriers to access led to dissatisfaction, which was associated with nonadherence. This result can be explained by the perceived barriers dimension of the health beliefs model, in which more barriers indicate greater dissatisfaction and lower adherence^39^. The perceived barriers and perceived benefits dimensions have consistently been shown to be the strongest predictors of health behaviors^36^.

The ACCO dimension had the lowest dissatisfaction rates in the study. This result may show that the services and products meet users’ expectations and that they feel satisfied. However, it is necessary to consider some factors that influence the mostly positive pattern of satisfaction evaluations, such as users being uncritical of the quality of the services they receive, whether due to lack of interest, lack of comparison models or a view of themselves as an actor in the process, and *gratitude* bias, which is more present in public health services and peripheral populations^5^.

When looking at the variables in the AVAI and ACCO dimensions, which showed higher rates of dissatisfaction, with the exception of those related to lack of access (already discussed above), we highlight problems with home use (difficulty reading the packaging and remembering to take it) and the moment of dispensing (dissatisfaction with the privacy of the service, receiving information about use, how to store the medicines at home and the comfort of the environment).

These results are a warning as to where patients’ difficulties and dissatisfaction lie, and their solutions involve patient-centered PS, such as quality dispensing and information by a trained professional in an appropriate environment for health care, clinical monitoring of medication use, training in self-care skills for CNCDs, which is still under construction in Brazil^40^.

Recent evaluations of the implementation, fidelity, and performance of the PS in the SUS provide a picture of great regional differences, activities focused mainly on product logistics, with recurring problems of supply, structure, and insufficient pharmacists in dispensing and monitoring treatments, which leaves the use of medicines under the responsibility of patients^40^. This organization hinders co-responsibility between professionals/services and patients in the management of diseases, the monitoring of patients taking medication, adherence to treatments, and the achievement of better health outcomes.

Measuring dissatisfaction using the IRT with closed questions had limitations because it did not directly address users’ beliefs, lifestyles, conceptions of health and illness, and expectations. However, a positive factor was the reduction in gratitude bias and other social factors that influence direct satisfaction surveys^12^. Other limitations of the study are the cross-sectional nature of the database, the non-use of all the adjustment variables for dissatisfaction and adherence described in the literature due to unavailability in the database, the difficulty of accessing data that should be public, the use of self-report and specific measures of nonadherence, the temporality of the data (2013–2014) and the effect of the lack of medication on the NAL and NA7 outcomes.

The limitations were mitigated by the use of a short recall period (seven days) for one of the outcomes, by the relationship between factor and outcome not being time-dependent, by the relevance of the PNAUM data, the largest survey on medication use in Brazil, and still unique in its relevance, the lack of studies in Brazil evaluating the association between dissatisfaction with health services and nonadherence to the use of medicines using theoretical and methodological models built for this purpose, and the heterogeneous characteristics of the pharmaceutical services in SUS PHC, which means that many Brazilian municipalities are now living the 2014 PNAUM reality. In addition, the organization of Esher’s model^11^helped to visualize the diversity of intermediate aspects that permeate the users-health services relationship, making it possible to investigate how users have different connections with dispensing units and the dimensions that influence satisfaction/dissatisfaction.

## FINAL CONSIDERATIONS

Most PHC users were satisfied with the PS, but those who were dissatisfied were less adherent to medication use. Dissatisfaction with the existence of services at the location and time required for users’ health (timeliness), and with the difference between the products/services offered and the users’ expectations and needs (accommodation), was associated with nonadherence to medication use.

More studies are needed on medication adherence in NCD patients in Brazil, and especially in the SUS, with longitudinal data collection and more in-depth analysis of the causes. In order to tackle the problem, it is necessary to understand it and thus propose ways forward according to the major causes and the reality of Brazilian health services.

In order to mitigate the dissatisfaction that influences nonadherence and is based on the difficulty of obtaining medicines, the lack of services that meet users’ expectations and needs in a timely manner, Brazil needs to strengthen public policies on access to medicines, implement and promote patient-centered PS, considering their needs and perspectives on the services received with management, dispensing and clinical follow-up activities carried out by pharmacists. Recent initiatives such as the (re)implementation of e-multi teams with a workload for pharmacists^45^and the publication of the National Guidelines for Pharmaceutical Care within the SUS^10^demonstrate steps in the construction of PS in the search for positive health outcomes.

## Data Availability

The data are available from the corresponding author upon request.
