# Supplementary material for: Dissatisfaction with SUS pharmaceutical services and nonadherence to medication use
Source: Rev Saude Publica. 2025 Dec 1;59:e36. doi: 10.11606/s1518-8787.2025059006463 (PMC12673644; doi:10.11606/s1518-8787.2025059006463)
Supplement: Translation

# Translation: Insatisfação com os serviços farmacêuticos do SUS e não adesão ao uso de medicamentos

**Authors:** Poliana Vieira da Silva Menolli, Edmarlon Girotto

**Affiliations:** I Universidade Estadual do Oeste do Paraná. Centro de Ciências Médicas e Farmacêuticas. Programa de Pós-Graduação em Ciências Farmacêuticas. Cascavel, PR, Brasil; II Universidade Estadual de Londrina. Centro de Ciências da Saúde. Departamento de Ciências Farmacêuticas. Programa de Pós-graduação em Saúde Coletiva. Londrina, PR, Brasil

**Keywords:** Satisfação do Paciente, Assistência Farmacêutica, Adesão à Medicação, Doença Crônica, Atenção Primária à Saúde

## Abstract

**OBJETIVO:**

Analisar a associação entre insatisfação dos usuários com os serviços farmacêuticos do Sistema Único de Saúde (SUS) e a não adesão ao uso de medicamentos por pacientes de doenças crônicas não transmissíveis.

**MÉTODOS:**

Estudo transversal com dados do componente de avaliação de serviços da Pesquisa Nacional de Acesso, Uso e Promoção do Uso Racional de Medicamentos no Brasil, realizada de julho a dezembro de 2014. Foram analisadas entrevistas presenciais de usuários do SUS de 18 anos ou mais que usavam medicamentos para hipertensão, diabetes e dislipidemia e as farmácias do SUS nos três meses anteriores à pesquisa. A variável dependente foi a não adesão aos medicamentos avaliada por meio de três medidas: não adesão por falta do medicamento, não adesão nos últimos sete dias e não adesão declarada. A variável independente foi a insatisfação com as dimensões dos serviços farmacêuticos (oportunidade, disponibilidade e adequação), obtida por meio da teoria de resposta ao item. A regressão logística foi realizada com cálculo dos *odds ratio* (OR) brutos e ajustados.

**RESULTADOS:**

Foram avaliados 2.448 usuários. A não adesão por falta foi de 31,6%, nos últimos sete dias de 11,5% e a declarada de 13,0% dos entrevistados. A falta de acesso e o esquecimento foram as principais justificativas para a não adesão nos últimos sete dias. Na análise ajustada a insatisfação com a dimensão “oportunidade” esteve associada à não adesão nos últimos sete dias (OR = 1,489; IC95% 1,023–2,168) e a dimensão “adequação” associada à não adesão declarada (OR = 3,132; IC95% 2,266–4,329).

**CONCLUSÃO:**

Os insatisfeitos foram mais não aderentes ao uso de medicamentos em todas as dimensões e a insatisfação com a disponibilidade e adequação dos serviços dos serviços farmacêuticos esteve associada à não adesão ao uso de medicamentos.

## INTRODUÇÃO

Para os usuários dos serviços de saúde, e em especial para os pacientes de doenças crônicas não transmissíveis (DCNT), a relação que se têm com os serviços e com os profissionais desses serviços é fator importante na construção dos julgamentos e crenças usados nas tomadas de decisão sobre os tratamentos e a motivação em manter os cuidados em saúde recomendados^1,2^.

Seguir o regime terapêutico como prescrito é chamado de “adesão ao tratamento”, que, segundo a Organização Mundial de Saúde (OMS), é a extensão na qual o comportamento de uma pessoa corresponde às recomendações acordadas com um profissional de saúde*,* orientações que vão de mudanças no estilo de vida ao uso de medicamentos, e que quando cumpridas levam a melhora clínica, redução de mortalidade e redução de custos em saúde^3^.

A satisfação é uma crença e uma expressão de atitude em relação a um processo de serviço, que na saúde se caracteriza pela avaliação subjetiva do paciente de sua reação cognitiva e emocional resultante da interação entre expectativas, experiências prévias e percepção do cuidado real recebido^4,5^.

A avaliação pode ser positiva (satisfação) ou negativa (insatisfação) e reflete como o paciente vê, vivência e julga os serviços de saúde recebidos^5^. Essa percepção e julgamento envolvem a experiência com os serviços, características dos próprios pacientes, como idade, suas representações sobre o processo saúde-doença, bem como o entendimento de seus direitos e de suas expectativas^1,6^.

Donabedian^7^ inclui a satisfação como resultado dos serviços de saúde e mesmo que haja uma discussão sobre sua capacidade para avaliar a qualidade dos serviços, não há dúvidas que traz valiosa informação da experiência em saúde na perspectiva do paciente^4^. Os usuários satisfeitos com os serviços de saúde tendem a ter melhor qualidade de vida, fornecem informações importantes ao provedor, voltam ao serviço quando necessário e são mais aderentes aos tratamentos prescritos^4,8^.

A consulta e os procedimentos médicos, as internações hospitalares e os cuidados de enfermagem são os serviços mais observados nas avaliações de satisfação dos usuários^1^, porém com a organização dos serviços nas redes de atenção à saúde e o foco no cuidado multiprofissional dos pacientes de DCNT, impera a necessidade de se conhecer e avaliar outros profissionais e serviços do itinerário dos pacientes^9^.

A assistência farmacêutica é parte do direito integral à saúde dos brasileiros e tem por objetivo o acesso qualificado, igualitário e universal aos medicamentos e a promoção do seu uso racional. É considerada uma ação estratégica dentro do cuidado em saúde no Sistema Único de Saúde (SUS) e hoje se organiza para além de seu ciclo inicial com foco no produto, com uma construção de serviços farmacêuticos (SF) que buscam estar centrados nos pacientes^10^. Os SF podem ser técnico-gerenciais, com atividades de gestão de produtos e serviços que apoiam as redes de atenção à saúde e técnico-assistenciais, com cuidados diretos aos pacientes e comunidades, que visam melhorar os resultados clínicos, promover a adesão aos tratamentos e prevenir eventos adversos^10^. Serviços farmacêuticos acessíveis e de qualidade promovem a satisfação do usuário^11,12^.

A adesão ao uso de medicamentos não é fácil de se medir, não há um padrão ouro para se comparar e o grande número de métodos e interpretações das medidas dificultam um consenso^13^. A adesão/não adesão é um evento complexo e dinâmico, que varia no tempo, de acordo com o tipo de doença e o número de medicamentos^14^, por isso modernamente se acredita na avaliação da história da dosificação de cada medicamento para resultados mais precisos^15^. Medidas feitas num ponto estático no tempo e por autorrelato, mesmo criticadas, são ainda o único método ao alcance de todos os profissionais de saúde, e são capazes de sinalizar e avaliar componentes importantes da adesão^14^.

No Brasil, são poucos os estudos que avaliam a relação entre satisfação do usuário com os serviços de saúde e a não adesão ao uso de medicamentos^16^ e não há nenhum, até o momento, que avalie a insatisfação com os SF e sua influência na não adesão exclusivamente. Nesse contexto, o objetivo do trabalho foi analisar a associação entre insatisfação dos usuários com SF do SUS e a não adesão ao uso de medicamentos por pacientes de DCNT que utilizavam medicamentos para hipertensão, diabetes e dislipidemia.

## MÉTODOS

Este é um estudo transversal, com dados da Pesquisa Nacional sobre Acesso, Utilização e Promoção do Uso Racional de Medicamentos (PNAUM) componente serviços, que teve por objetivo caracterizar a organização dos serviços farmacêuticos na atenção primária à saúde (APS) do SUS em relação ao acesso e à promoção do uso racional de medicamentos, e identificar e discutir os fatores que interferem na consolidação dos SF no âmbito municipal. Os dados foram coletados de julho a dezembro de 2014 e detalhes da metodologia da PNAUM Serviços, bem como o processo amostral, estão descritos em Álvares et al.^19^

A PNAUM Serviços entrevistou 8.803 usuários do SUS maiores de 17 anos, que estiveram nos serviços de APS à espera de uma consulta médica, capazes de responder às questões propostas e concordaram em participar da pesquisa.

Este estudo seguiu as diretrizes de *The Strengthening the Reporting of Observational Studies in Epidemiology* (STROBE)^20^ e analisou as entrevistas presenciais realizadas com os usuários da APS do SUS de 18 anos ou mais que declararam usar medicamentos para tratamento da hipertensão arterial sistêmica (HAS), diabetes e dislipidemia e que usaram as farmácias do SUS nos três meses anteriores à pesquisa. Esse recorte foi escolhido por serem tratamentos de curso longo, já iniciados anteriormente, que fazem do paciente um ator experiente no contato com os SF e para os quais a não adesão aos tratamentos caracteriza um problema importante para pacientes e serviços de saúde^3^.

Foram considerados como tratamento os grupos terapêuticos da *Anatomical Therapeutic Chemical Classification* da OMS: para diabetes os medicamentos pertencentes ao grupo terapêutico A10 (medicamentos usados na diabetes); para a HAS os medicamentos dos grupos terapêuticos C2 (anti-hipertensivos), C3 (diuréticos), C4 (vasodilatadores periféricos), C7 (agentes beta-bloqueadores), C8 (bloqueadores de canais de cálcio), C9 (agentes que atuam no sistema renina-angiotensina); e para dislipidemias o grupo terapêutico C10 (agentes modificadores de lipídeos)^21^.

As questões do instrumento sobre a satisfação do usuário foram baseadas no *Long-Form Patient Satisfaction Questionnaire* (PSQ-III), adaptado para os serviços farmacêuticos, segundo Soeiro et al.^12^

Todos os bancos de dados da PNAUM são públicos, mas não estão mais disponíveis publicamente, sendo preciso solicitar acesso ao Ministério da Saúde. O banco avaliação de serviços para esse estudo foi obtido no ano de 2020.

### Variáveis

Os entrevistados foram caracterizados segundo: região do Brasil (Norte, Nordeste, Centro-Oeste, Sul e Sudeste), sexo (feminino e masculino), etnia (branca/amarela e negra/parda/indígena), situação conjugal (com companheiro e sem companheiro), escolaridade (0 a 8 anos e acima de 8 anos de estudo), idade (17 a 39, 40 a 59 e igual ou maior que 60 anos), número de doenças por autorrelato (1 a 5 e acima de 5), polifarmácia – uso de 4 medicamentos ou mais (sim e não), necessidade de ajuda para tomar os medicamentos (sim e não), atendimento de emergência nos últimos 12 meses (sim e não) e internação hospitalar nos últimos 12 meses (sim e não).

### Avaliação da Não Adesão ao Uso de Medicamentos

A não adesão ao tratamento foi avaliada de maneira pontual por meio de três variáveis que sinalizavam o comportamento de deixar de usar os medicamentos prescritos: não adesão por falta do medicamento (NAF), não adesão nos últimos sete dias (NA7) e não adesão declarada (NAD).

A NAF avaliou a dificuldade de se obter o medicamento, apontando de maneira indireta o não uso dos medicamentos, e foi construída a partir da pergunta: “*Você teve algum problema para conseguir esse remédio*?” que admitia respostas “sim” e “não”. A NA7 avaliou o não uso do medicamento na semana anterior e foi construída a partir da pergunta: “*Você deixou de tomar este remédio, por algum motivo, nos últimos 7 dias*?” que admitia respostas “sim” e “não”. A variável NAD avaliou a não adesão declarada e/ou intencional, quando o paciente declara que por vontade própria deixa de tomar o medicamento, por meio da pergunta: “*Você deixa de utilizar algum remédio receitado pelo médico?”* com respostas “sim” e “não”.

As variáveis NAF e NA7 foram construídas a partir de perguntas para cada medicamento usado pelo entrevistado. Nas perguntas por medicamento as respostas “sim” (problema em conseguir e deixar de tomar) ganharam valor 1 e as “não” valor 0. Os valores foram somados e divididos pelo total de medicamentos usados pelo paciente. Foi considerada não adesão para as variáveis NAF e NA7 resultado maior que 0,2 (mais que 20% dos medicamentos com problemas em serem obtidos ou deixados de serem tomados), valor baseado no conceito de adesão de Sackett et al.^22^ e OMS^3^. Na NA7 também foram observados os motivos declarados pelo paciente para ter deixado de tomar na semana anterior. A NAD foi construída por meio de uma única pergunta por entrevistado, com as respostas “sim” consideradas não adesão.

As variáveis foram avaliadas isoladamente, ou seja, um mesmo paciente pode ser não aderente nas três medidas.

### Avaliação da Insatisfação do Usuário

A avaliação da insatisfação do usuário com os SF foi feita por meio do modelo teórico desenvolvido por Esher^11^ para avaliação da satisfação do usuário com a dispensação de medicamentos para HIV, adaptado para avaliação dos SF^12^.

Nesse modelo, a satisfação é mediada pelo acesso (relação entre obstáculos encontrados pelo usuário na busca pela atenção em saúde e a capacidade da população de superar tais obstáculos), assim mais acesso é considerado satisfação e menos, insatisfação, o que torna a insatisfação o oposto direto da satisfação. Pelo acesso é possível avaliar operacionalmente a dispensação e os serviços farmacêuticos, pois problemas nas dimensões do acesso aos serviços e produtos de saúde influenciam os usuários e o próprio sistema, modificando a satisfação do usuário e mudando os padrões de organização e utilização dos serviços^11^.

Por considerar a dispensação uma etapa chave dos SF, em que todas as etapas anteriores são efetivamente colocadas na rotina de cuidados em saúde dos pacientes e por ser a etapa que é o elo para o acompanhamento do uso de medicamentos, esta pesquisa utilizou o modelo de avaliação da satisfação do usuário criado por Esher^11^ para a avaliação da insatisfação com os SF na APS do SUS.

Na análise, foram usadas as seguintes dimensões do acesso: oportunidade/conveniência (OP/CO), disponibilidade (DISP) e adequação (AD), que são os critérios usados pelo usuário para julgar a satisfação com foco na experiência corrente com o serviço e não no efeito dele na saúde. A dimensão AD foi dividida nas subdimensões qualidade técnica da dispensação, qualidade técnica do medicamento, ambiência e aspectos interpessoais^11,12^.

As variáveis-resposta foram agrupadas nas três dimensões: OP/CO, DISP e AD, e dicotomizadas, considerando “0” como satisfeito e “1” como insatisfeito (Quadro).

**Quadro:** Constructo das variáveis relacionadas à insatisfação do usuário com os serviços farmacêuticos.

Dimensão	Subdimensão	Variável	Perguntas
**Oportunidade** Existência dos serviços no lugar e no momento em que tecnicamente é requerido pela situação de saúde dos usuários.	**Conveniência** Distância/facilidade de acesso geográfico, organização do serviço, tempo despendido pelo usuário e possibilidade de escolha de unidades de saúde	Distância do domicílio	*Este lugar é longe da sua casa?* (Assumiu valor 0 para a resposta “não” e 1 para as respostas “sim” e “mais ou menos”).
		Dificuldade para chegar ao serviço	*Chegar aqui é* (“Muito fácil” e “Fácil” receberam código 0, as respostas “Nem fácil/nem difícil”, “Difícil” e “Muito difícil” receberam código 1*)*.
		Horário de funcionamento	*O horário de funcionamento desta Unidade de Saúde é*: (“Muito bom” e “Bom” receberam código 0, as respostas “Nem ruim/nem bom”, “Ruim” e “Muito ruim” receberam código 1*)*.
		Tempo de espera	*Normalmente, quanto tempo você espera para retirar os remédios nas farmácias públicas do SUS?* (As respostas “Não espera” e “Um pouco” receberam código 0 e a resposta “Muito tempo” recebeu 1*)*.
**Disponibilidade** Relação entre o tipo e a quantidade de produtos e serviços necessários e o tipo e a quantidade de serviços oferecidos	**Presença do medicamento** Disponibilidade do medicamento na farmácia na quantidade prescrita para o usuário	Conseguiu medicamento gratuito	*Onde conseguiu este remédio pela última vez?* (Assumiu código 0 para as respostas “Farmácia do SUS”, “Farmácia Popular” ou “Igreja ou sindicato” (que estaria relacionado a uma facilitação do acesso ao medicamento, promovendo satisfação) e 1 para a resposta “Farmácia comercial”). No caso dos pacientes que tomaram mais de um medicamento, se houve algum medicamento para o qual ele deixou de tomar, a resposta dele seria 1*)*.
		Problema para conseguir	*Teve algum problema para conseguir remédio da última vez?* (Assumiu código 0 para a resposta “não teve problema” e 1 para as respostas que indicavam que o paciente teve problema para conseguir o remédio). No caso dos pacientes que tomaram mais de um medicamento, se houve algum medicamento para o qual ele deixou de tomar, a resposta dele seria 1*)*.
		Deixou de tomar nos últimos sete dias	*Você deixou de tomar o medicamento por algum motivo nos últimos sete dias?* (Assumiu valor 0 para o não e 1 para o sim). No caso dos pacientes que tomaram mais de um medicamento, se houve algum medicamento para o qual ele deixou de tomar, a resposta dele seria 1*)*.
		Medicamentos no SUS nos últimos três meses	*Nos últimos três meses, com que frequência você conseguiu os remédios que procurava nas farmácias públicas do SUS?* (As respostas “sempre” e “repetidamente” receberam código 0, as respostas “às vezes”, “raramente” e “nunca” receberam código 1*)*.
		Dificuldades com o medicamento de 1 a 6	*1. Teve dificuldade de lembrar de tomar o remédio? 2. Teve dificuldade para lidar com remédios por que utiliza muitos comprimidos ao dia? 3. Teve dificuldade para lidar com remédios por que é difícil consegui-los? 4. Teve dificuldade para lidar com remédios por que é difícil ler o que está escrito na embalagem? 5. Teve dificuldade para lidar com remédios por que é difícil adequar a utilização deles com o seu trabalho? 6. Teve dificuldade para lidar com remédios por que há remédios diferentes com a mesma forma e coloração?* A resposta “não” recebeu código 0 e a resposta “sim” código 1.
**Adequação** Ajuste entre as características dos produtos e serviços e as expectativas e necessidades dos usuários	**Qualidade técnica da dispensação** Competência de provedores e à adesão aos componentes essenciais da dispensação.	Recebe informação	*Quando você retira remédios nas farmácias públicas do SUS, os funcionários que entregam os remédios repassam informações sobre a utilização dos mesmos?* Resposta “sim” recebeu código 0 e as respostas “às vezes” e “não” código 1.
		Entende a informação	*Você entende as informações repassadas pelos funcionários que entregam remédios nas farmácias públicas do SUS?* As respostas “sempre” e “repetidamente” receberam código 0 e as respostas “às vezes”, “raramente” e “nunca” código 1.
		Farmacêutico/ Funcionário disponível	*O farmacêutico ou outro funcionário da farmácia pública do SUS está disponível quando você precisa tirar dúvidas sobre os remédios?* As respostas “sempre” e “repetidamente” receberam código 0 e as respostas “às vezes”, “raramente” e “nunca” código 1.
		Orientação como guardar em casa	*Quando retira remédios nas farmácias públicas do SUS, você recebe orientação sobre como guardá-los em casa?* As respostas “Sempre” e “repetidamente” receberam código 0, as respostas “às vezes”, “raramente” e “nunca” receberam código 1.
	**Qualidade técnica do medicamento** Características das embalagens e à apresentação dos medicamentos, à produção de reações adversas e à possível resolução de sintomas.	Efeito SUS x comercial	*Para você os efeitos dos remédios recebidos nas farmácias pública do SUS em comparação com os efeitos dos remédios comprados em farmácia comercial são*: As respostas “iguais” e “melhores” receberam código 0 e a resposta “piores” código 1.
		Efeito do medicamento	*Como esse remédio está funcionando para sua doença?* Assumiu código 0 para a resposta “bem” e 1 para as respostas “regular” e “não funciona bem”. No caso dos pacientes que tomaram mais de um medicamento, se houve algum medicamento que ele julgou funcionar de forma regular ou não funcionar bem, a resposta dele seria 1.
		Causa problema de saúde	*Em sua opinião, este remédio causa algum problema de saúde para você?* Assumiu código 0 para a resposta “não” e 1 para a resposta “sim”. No caso dos pacientes que tomaram mais de um medicamento, se houve algum medicamento que causou problema, a resposta dele seria 1.
	**Ambiência** Características do local onde o cuidado é fornecido e deve garantir adequação das instalações às condições mínimas para um ambiente confortável (limpeza, sinalização, ventilação suficiente, lugar protegido de intempéries, água limpa para beber)	Sinalização	*Como você avalia a sinalização para encontrar a farmácia pública do SUS?* As respostas “muito fácil” e “fácil” receberam código 0 e as respostas “nem fácil/nem difícil”, “difícil” e “muito difícil” código 1.
		Limpeza	*Como você avalia a limpeza da farmácia pública do SUS?* As respostas “muito boa” e “boa” receberam código 0 e as respostas “nem ruim/nem boa”, “ruim” e “muito ruim” código 1.
		Conforto	*Como você avalia o conforto da farmácia pública do SUS?* As respostas “muito bom” e “bom” receberam código 0 e as respostas “nem ruim/nem bom”, “ruim” e “muito ruim” código 1.
		Atendimento	*Como você avalia o atendimento da farmácia pública do SUS?* As respostas “muito bom” e “bom” receberam código 0 e as respostas “nem ruim/nem bom”, “ruim” e “muito ruim” código 1.
	**Relações interpessoais** Atitude dos dispensadores em relação aos usuários no momento do fornecimento dos medicamentos e inclui os componentes de autonomia, dignidade e confidencialidade	Respeito/cortesia	*Os funcionários da farmácia pública do SUS atendem com respeito e cortesia?* As respostas “sempre” e “repetidamente” receberam código 0 e as respostas “às vezes”, “raramente” e “nunca” código 1.
		Privacidade	*Você considera que o atendimento na farmácia pública do SUS ocorre com privacidade?* As respostas “sempre” e “repetidamente” receberam código 0 e as respostas “às vezes”, “raramente” e “nunca” código 1.
		Avaliação atendimento	*Como você avalia o atendimento da farmácia pública do SUS?* As respostas “muito bom” e “bom” receberam código 0 e as respostas “nem ruim/nem bom”, “ruim” e “muito ruim” código 1.

As variáveis das dimensões OP/CO e AD foram construídas a partir de uma única pergunta por entrevistado. Na dimensão DISP as variáveis foram construídas por meio de perguntas por medicamento usado, assim os valores para insatisfação (1) em cada pergunta foram somados e divididos pelo número de medicamentos. Resultados iguais ou acima de 0,5 foram considerados insatisfeitos e classificados como “1” e valores menores que 0,5 satisfeitos e classificados como “0”.

Para que a insatisfação do usuário fosse representada por uma variável latente de cada dimensão, originalmente não observada, foi utilizada a técnica de teoria de resposta ao item (TRI)^23^. O índice de insatisfação por dimensão foi criado e transformado em uma escala de 0 a 1, em que a proximidade com o 0 indicava maior satisfação e com o 1, maior insatisfação.

Para dicotomizar as respostas de cada uma das variáveis, utilizou-se a mediana, sendo que valores maiores ou iguais assumiriam o código 1 (insatisfeito) e menores valor 0 (satisfeito). Os modelos TRI foram construídos no programa R versão 4.1.2, utilizando o pacote *lt* ^23^. O nível de significância adotado foi de 5%.

## Análise dos Dados

Os dados foram tratados no programa Microsoft Excel, exportados e analisados com o uso do IBM *Statistical Package for the Social Sciences*® (IBM-SPSS) versão 19.0, com o uso dos comandos CSPLAN, apropriado para amostras complexas^19^, que considera pesos amostrais e efeitos do desenho. A análise descritiva foi realizada por meio de apresentação de frequências relativas e intervalos de confiança das frequências relativas. A associação entre as variáveis de ajuste, escolhidas por terem influência na adesão^24^, e os três desfechos foi medida pelo teste *X* ^2^ de Pearson. Por fim, depois de descartar o efeito de interação das variáveis independentes com as de ajuste nas medidas de adesão ao tratamento, conduziu-se as análises de regressão.

Para a análise da relação entre insatisfação dos usuários e não adesão ao tratamento, foi realizada a regressão logística bruta e ajustada e os resultados apresentados por meio de razão de chances (*odds ratio* [OR]). O ajuste do modelo foi feito com as variáveis que obtiveram resultado de p < 0,20 na análise de associação (teste *X* ^2^ de Pearson), independente para cada desfecho testado. A variável sexo foi incluída em todos os modelos de ajuste independentemente do valor de p na análise de associação. O nível de significância estatística estabelecido neste estudo foi de 5% (p < 0,05).

A PNAUM foi aprovada pela Comissão Nacional de Ética em Pesquisa (CONEP) por meio do Parecer nº 398.131, de 16 de setembro de 2013. As entrevistas foram realizadas após explicação dos objetivos do estudo e concordância por parte do entrevistado ou responsável legal, com assinatura de termo de consentimento livre e esclarecido.

## RESULTADOS

Dos 8.803 entrevistados pela PNAUM Serviços, foram excluídos 2.303 que não usavam medicamentos, 3.567 que usavam medicamentos que não eram tratamento para diabetes, HAS e dislipidemia, 450 entrevistados por não terem utilizado as farmácias do SUS nos três meses anteriores à pesquisa e 35 por responderem “não sei” para as perguntas de adesão ao uso de medicamentos (desfecho). Assim, foram avaliados 2.448 usuários.

A maioria era mulher (73,9%), acima dos 60 anos (47,5%), negra, parda ou indígena (57,0%), com companheiro(a) (67,8%), com até oito anos de estudo (80,5%), declarou ter até cinco doenças (88,3%) e utilizar de um a três medicamentos (68,4%), não precisava de ajuda para tomar os medicamentos (90,1%) e era moradora das regiões Sudeste (38,5%) e Sul (26,1%) (Tabela 1).

**Tabela 1:** Caracterização dos pacientes em tratamento para hipertensão arterial sistêmica, diabetes e dislipidemias atendidos pelos serviços farmacêuticos do componente avaliação de serviços da PNAUM, 2014, (n = 2.448 pessoas).

Variáveis de caracterização e ajuste	Total (%)	Não adesão por falta de acesso		Não adesão 7 dias		Não adesão declarada	
		%	p^a^	%	p^a^	%	p^a^
Região do Brasil			0,110		0,643		0,214
Norte	3,6	41,3		13,0		10,8	
Nordeste	27,3	35,4		10,4		14,9	
Centro-Oeste	4,3	34,0		14,7		12,9	
Sul	26,1	26,3		12,3		14,2	
Sudeste	38,5	31,2		11,3		11,0	
Sexo			0,680		0,022		0,451
Feminino	73,9	32,9		12,6		12,6	
Masculino	26,1	27,7		8,4		14,1	
Etnia			0,509		0,918		0,266
Branca/amarela	43,0	32,4		11,5		14,1	
Negra/parda/indígena	57,0	30,7		11,6		12,0	
Situação conjugal			0,328		0,601		0,718
Com companheiro	67,8	32,4		11,8		13,2	
Sem companheiro	32,2	29,8		10,9		12,5	
Escolaridade			0,003		< 0,001		0,008
0 a 8 anos	80,5	29,8		10,0		14,1	
Acima de 8 anos	19,5	38,9		17,7		8,3	
Idade (em anos)			0,544		< 0,001		0,145
17 a 39	9,3	29,0		18,9		18,3	
40 a 59	45,0	33,0		14,6		11,8	
≥ 60 anos	45,7	30,7		7,0		13,0	
Número de doenças			0,443		0,996		0,571
0 ou não sabe	8,2	34,4		11,4		10,6	
1 a 5	88,3	31,0		11,5		13,3	
Acima de 5	3,5	38,0		11,9		10,5	
Polifarmácia			< 0,001		0,274		0,353
Sim	31,6	39,8		12,9		11,8	
Não	68,4	27,8		10,9		13,5	
Precisa ajuda para tomar medicamentos			0,151		0,181		0,739
Sim	9,9	37,1		8,3		13,9	
Não	90,1	31,0		11,9		12,9	
Atendimento de emergência (últimos 12 meses)			0,003		< 0,001		0,020
Sim	23,5	38,0		18,4		9,4	
Não	76,5	29,6		9,4		14,1	
Internação (últimos 12 meses)			0,090		0,010		0,054
Sim	11,5	37,3		17,6		8,7	
Não	88,5	30,8		10,7		13,5	

PNAUM: Pesquisa Nacional sobre Acesso, Utilização e Promoção do Uso Racional de Medicamentos.

^a^ Teste *X* ^2^ de Pearson.

A NAF foi identificada em 31,6%, a NA7 em 11,5% e a NAD em 13,0% dos entrevistados. As justificativas para o não uso na semana anterior foram estar sem o medicamento (sem receita, sem consulta, em falta na unidade básica de saúde, em falta na farmácia e sem dinheiro para comprar) (50,2%), seguido do esquecimento (22,9%), presença de efeitos indesejados (9,3%), iniciativa própria (7,3%), entre outras.

A NAF foi superior nos usuários com maior escolaridade, que referiram polifarmácia, e que receberam atendimento de emergência ou referiram internação nos últimos 12 meses. A NA7 apresentou maior prevalência nas mulheres, em usuários com maior escolaridade e menor idade, e nos que receberam atendimento de emergência ou referiram internação nos últimos 12 meses. Já a NAD demonstrou-se maior nos usuários de menor escolaridade e que não receberam atendimento de emergência ou referiram internação nos últimos 12 meses (Tabela 1).

Os usuários estavam em sua maioria satisfeitos com os serviços farmacêuticos de APS dos municípios e as maiores taxas de insatisfação foram encontradas nas varáveis *dificuldade de ler a embalagem*, na dimensão Disponibilidade (DISP), com 41,8% de insatisfeitos, seguida da *distância do domicílio*, na dimensão oportunidade/conveniência (OP/CO), com 38,6% de insatisfeitos e *conseguiu os medicamentos no SUS*, com 36,5% de insatisfação (Tabela 2).

**Tabela 2:** Prevalência da não adesão ao uso de medicamentos segundo as variáveis de insatisfação dos usuários atendidos pelos SF da APS, PNAUM, 2014, (n = 2.448).

Dimensão/subdimensão da insatisfação	Prevalência % (IC95%)			
	Total %	Não adesão por falta	Não adesão 7 dias	Não adesão declarada
Oportunidade/conveniência				
Distância do domicílio				
Satisfeitos	61,4 (58,8–63,9)	31,5 (28,5–34,7)	10,2 (8,5–12,3)	12,9 (10,8–15,3)
Insatisfeitos	38,6 (36,1–41,2)	31,6 (27,7–35,7)	13,4 (10,9–16,8)	13,1 (10,4–16,3)
Dificuldade para chegar ao serviço				
Satisfeitos	82,4 (80,2–84,3)	30,7 (28,1–33,4)	10,2 (8,7–12,0)	12,9 (11,1–15,0)
Insatisfeitos	17,6 (15,7–19,8)	35,5 (29,7–41,8)	17,5 (13,2–22,9)	13,4 (9,6–18,4)
Horário de funcionamento				
Satisfeitos	89,7 (88,0–91,2)	30,2 (27,7–32,8)	10,8 (9,2–12,6)	12,8 (11,0–14,8)
Insatisfeitos	10,3 (8,8–12,0)	43,2 (35,3–51,4)	18,3 (12,8–25,4)	15,1 (10,1–22,0)
Tempo de espera				
Satisfeitos	94,8 (93,5–95,9)	30,9 (28,5–33,5)	11,4 (9,8–13,2)	13,2 (11,5–15,2)
Insatisfeitos	5,2 (4,1–6,5)	43,8 (32,7–55,5)	12,8 (6,9–22,5)	9,4 (4,7–17,8)
Disponibilidade/presença do medicamento				
Conseguiu os medicamentos no SUS				
Satisfeitos	63,5 (61,0–65,9)	13,1 (11,0–15,5)	7,5 (6,0–9,4)	14,3 (12,1–16,8)
Insatisfeitos	36,5 (34,1–39,0)	63,7 (59,5–67,6)	18,5 (15,4–22,1)	10,7 (8,3–13,6)
Problema para conseguir				
Satisfeitos	64,5 (62,0–67,0)	-	9,0 (7,4–11,0)	13,5 (11,4–16,0)
Insatisfeitos	35,5 (33,0–38,0)	89,0 (86,1–91,4)	16,1 (13,1–19,5)	12,0 (9,4–15,1)
Deixou de tomar nos últimos sete dias				
Satisfeitos	85,5 (83,5–87,3)	31,9 (29,3–34,6)	10,9 (9,3–12,8)	9,7 (8,1–11,5)
Insatisfeitos	14,5 (12,7–16,5)	29,7 (23,7–36,5)	15,0 (10,7–20,6)	32,1 (26,0–39,0)
Medicamentos no SUS nos últimos três meses				
Satisfeitos	73,8 (71,5–76,1)	33,4 (30,6–36,4)	10,6 (8,9–12,6)	11,2 (9,4–13,3)
Insatisfeitos	26,2 (23,9–28,5)	23,6 (22,1–31,0)	14,0 (10,9–18,0)	17,9 (14,3–22,1)
Disponibilidade/presença do medicamento/ dificuldades com o medicamento				
Teve dificuldade de lembrar de tomar o remédio?				
Satisfeitos	71,2 (68,8–73,5)	31,4 (28,6–34,4)	11,5 (9,7–13,6)	13,3 (11,3–15,6)
Insatisfeitos	28,8 (26,5–31,2)	31,9 (27,6–36,5)	11,6 (8,9–14,9)	12,1 (9,3–15,5)
Teve dificuldade para lidar com remédios por que utiliza muitos comprimidos ao dia?				
Satisfeitos	77,7 (75,5–79,8)	30,8 (28,1–33,7)	11,5 (9,7–13,5)	14,3 (12,3–16,6)
Insatisfeitos	22,3 (20,2–24,5)	34,4 (29,5–39,7)	10,8 (7,9–14,5)	8,4 (6,0–11,6)
Teve dificuldade para lidar com remédios por que é difícil consegui-los?				
Satisfeitos	73,4 (71,1–75,6)	25,5 (22,9–28,3)	10,2 (8,5–12,1)	14,0 (11,9–16,3)
Insatisfeitos	26,6 (24,4–28,9)	48,3 (43,4–53,2)	15,3 (12,0–19,2)	10,2 (7,6–13,4)
Teve dificuldade para lidar com remédios por que é difícil ler o que está escrito na embalagem?				
Satisfeitos	58,2 (55,6–60,8)	32,3 (29,2–35,6)	10,1 (8,3–12,3)	12,4 (10,2–14,8)
Insatisfeitos	41,8 (39,2–44,4)	30,5 (26,8–34,4)	13,5 (11,0–16,5)	13,9 (11,3–17,0)
Teve dificuldade para lidar com remédios por que é difícil adequar a utilização deles com o seu trabalho?				
Satisfeitos	85,9 (84,1–87,6)	31,1 (28,6–33,8)	11,5 (9,8–13,4)	13,4 (11,6–15,5)
Insatisfeitos	14,1 (12,4–15,9)	32,4 (26,4–38,9)	11,9 (8,4–16,6)	10,7 (7,3–15,4)
Teve dificuldade para lidar com remédios por que há remédios diferentes com a mesma forma e coloração?				
Satisfeitos	81,5 (79,5–83,4)	30,8 (28,1–33,5)	12,0 (10,2–14,0)	13,2 (11,3–15,4)
Insatisfeitos	18,5 (16,6–20,5)	35,5 (30,0–41,3)	9,3 (6,6–13,0)	12,3 (8,9–16,7)
Adequação/ qualidade técnica da dispensação				
Recebe informação				
Satisfeitos	72,8 (70,4–75,1)	32,0 (29,2–35,0)	11,1 (9,4–13,2)	9,7 (8,0–11,7)
Insatisfeitos	27,2 (24,9–29,6)	30,7 (26,3–35,5)	12,7 (9,8–16,3)	21,6 (17,7–26,1)
Entende a informação				
Satisfeitos	94,9 (93,5–96,0)	31,4 (28,7–34,3)	11,2 (9,4–13,2)	10,3 (8,5–12,3)
Insatisfeitos	5,1 (4,0–6,5)	35,7 (24,6–48,5)	15,4 (8,4–26,6)	13,5 (6,9–24,8)
Farmacêutico/Funcionário disponível				
Satisfeitos	82,5 (80,4–84,5)	31,9 (29,2–34,8)	11,0 (9,4–13,0)	10,2 (8,5–12,2)
Insatisfeitos	17,5 (15,5–19,6)	31,4 (25,8–37,7)	12,4 (8,8–17,2)	19,8 (15,1–25,5)
Orientação como guardar em casa				
Satisfeitos	74,8 (72,2–77,2)	32,2 (29,0–35,4)	11,2 (9,2–13,5)	8,6 (6,8–10,7)
Insatisfeitos	25,2 (22,8–27,8)	30,2 (25,2–35,8)	12,3 (9,1–16,4)	16,9 (13,0–21,6)
Adequação/ qualidade técnica do medicamento				
Efeito SUS x comercial				
Satisfeitos	92,8 (91,1–94,1)	30,6 (28,0–33,4)	11,6 (9,9–13,5)	11,0 (9,3–13,0)
Insatisfeitos	7,2 (5,9–8,9)	31,0 (22,0–41,7)	16,7 (10,0–26,6)	29,0 (20,3–39,6)
Efeito do medicamento				
Satisfeitos	87,5 (85,7–89,2)	31,3 (28,7–33,9)	10,5 (9,0–12,3)	12,8 (11,0–14,8)
Insatisfeitos	12,5 (10,8–14,3)	33,6 (27,0–41,0)	18,6 (13,6–24,9)	14,1 (9,6–20,4)
Causa problema de saúde				
Satisfeitos	92,7 (91,2–93,9)	31,2 (28,7–33,8)	11,2 (9,6–13,0)	12,6 (10,9–14,5)
Insatisfeitos	7,3 (6,1–8,8)	36,4 (27,9–45,8)	15,5 (10,2–22,9)	17,7 (11,5–23,6)
Adequação/ ambiência				
Sinalização				
Satisfeitos	91,2 (89,6–92,6)	31,7 (29,2–34,3)	11,6 (10,0–13,4)	11,4 (9,8–13,3)
Insatisfeitos	8,8 (6,6–9,5)	30,3 (22,7–39,1	12,0 (7,2–19,5)	29,2 (21,7–38,1)
Limpeza				
Satisfeitos	92,0 (90,5–93,4)	31,5 (29,0–34,1)	11,1 (9,5–12,9)	11,0 (9,4–12,9)
Insatisfeitos	8,0 (6,6–9,5)	30,6 (22,5–40,0)	14,2 (8,7–22,4)	26,1 (18,5–35,5)
Conforto				
Satisfeitos	74,6 (72,2–76,8)	31,5 (28,7–34,4)	11,2 (9,5–13,2)	9,9 (8,2–11,9)
Insatisfeitos	25,4 (23,2–27,8)	32,5 (27,8–37,5)	12,9 (9,8–16,9)	21,7 (17,7–26,4)
Atendimento				
Satisfeitos	91,3 (89,7–92,7)	31,5 (29,0–34,1)	11,4 (9,8–13,2)	11,3 (9,7–13,2)
Insatisfeitos	8,7 (7,3–10,3	31,9 (24,1–40,8)	13,7 (8,6–21,1)	30,9 (23,2–39,8)
Adequação/ relações interpessoais				
Respeito/cortesia				
Satisfeitos	93,8 (92,4–94,9)	31,5 (29,1–39,1)	11,4 (9,8–13,2)	11,5 (9,8–13,3)
Insatisfeitos	6,2 (5,1–7,6)	33,1 (23,9–43,8)	13,9 (8,0–22,9)	35,6 (26,3–46,2)
Privacidade				
Satisfeitos	71,4 (68,9–73,8)	32,1 (29,2–35,1)	10,3 (8,6–12,4)	9,6 (7,9–11,7)
Insatisfeitos	28,6 (26,2–31,1)	30,9 (26,4–35,7)	13,9 (10,8–17,8)	20,2 (16,4–24,5)
Avaliação atendimento				
Satisfeitos	91,3 (89,7–92,7	31,5 (29,0–34,1)	11,4 (9,8–13,2)	11,3 (9,7–13,2)
Insatisfeitos	8,7 (7,3–10,3)	31,9 (24,1–40,8)	13,7 (8,6–21,1)	30,9 (23,2–39,8)

IC95%: intervalo de confiança de 95%; SUS: Sistema único de Saúde. APS: atenção primária à saúde; SF: serviços farmacêuticos; PNAUM: Pesquisa Nacional sobre Acesso, Utilização e Promoção do Uso Racional de Medicamentos.

A NAF foi maior em todas as dimensões estudadas, tanto entre os satisfeitos como entre os insatisfeitos, com destaque para a dimensão DISP que apresentou as maiores taxas. A NA7 foi maior entre os insatisfeitos em todas as dimensões. A NAD também foi maior entre os insatisfeitos, com destaque para a dimensão Adequação (AD), cujas taxas foram três vezes os valores observados para os satisfeitos (Tabela 2).

A dimensão OP/CO se mostrou associada à NA7 na análise bruta e ajustada (OR = 1,489; IC95% 1,023–2,168; p = 0,038). A dimensão adequação (AD) esteve associada à NAD na análise bruta, que se confirmou na ajustada (OR = 3,132; IC95% 2,266–4,329; p < 0,001). Os resultados da regressão logística entre as dimensões da satisfação e adesão são apresentados na Tabela 3.

**Tabela 3:** Regressão logística bruta e ajustada das dimensões da insatisfação com serviços farmacêuticos da APS e a não adesão ao uso de medicamentos para HAS, diabetes e dislipidemia, Brasil 2014.

Não adesão falta de acesso					
Dimensões	% (IC95%)	OR (IC95%)	p	OR_a_ (IC95%)^a^	p
Oportunidade					
Satisfeito	29,9 (26,7–33,3)	1,000		1,000	
Insatisfeito	33,4 (29,9–37,0)	1,313 (0,994–1,734)	0,055	1,260 (0,947–1,678)	0,113
Disponibilidade					
Satisfeito	24,5 (21,5–27,8)	1,000		1,000	
Insatisfeito	39,5 (35,9–43,2)	1,294 (1,000–1,674)	0,050	1,188 (0,911–1,549)	0,204
Adequação					
Satisfeito	31,3 (28,0–34,8)	1,000		1,000	
Insatisfeito	31,9 (28,4–35,5)	0,914 (0,706–1,184)	0,496	0,899 (0,689–1,173)	0,431
**Não adesão 7 dias**					
**Dimensões**	**% (IC95%)**	**OR (IC95%)**	**p**	**OR_a_ (IC95%)^b^**	**p**
Oportunidade					
Satisfeito	9,6 (7,7–11,8)	1,000		1,000	
Insatisfeito	13,6 (11,2–16,4)	1,673 (1,152–2,428)	0,007	1,489 (1,023–2,168)	0,038
Disponibilidade					
Satisfeito	9,2 (7,3–11,4)	1,000		1,000	
Insatisfeito	14,2 911,8–17,0)	0,963 (0,665–1,394)	0,841	0,923 (0,628–1,357)	0,684
Adequação					
Satisfeito	10,1 (8,2–12,5)	1,000		1,000	
Insatisfeito	13,1 (10,8–15,8)	1,142 (0,799–1,632)	0,466	1,204 (0,839–1,729)	0,314
**Não adesão declarada**					
**Dimensões**	**% (IC95%)**	**OR (IC95%)**	**p**	**OR_a_ (IC95%)^c^**	**p**
Oportunidade					
Satisfeito	12,8 (10,6–15,5)	1,000		1,000	
Insatisfeito	13,1 (10,7–15,9)	1,102 (0,745–1,630)	0,627	1,117 (0,751–1,661)	0,585
Disponibilidade					
Satisfeito	14,6 (12,1–17,4)	1,000		1,000	
Insatisfeito	11,2 (9,1–13,8)	0,789 (0,543–1,145)	0,212	0,815 (0,558–1,189)	0,288
Adequação					
Satisfeito	7,0 (5,3–9,1)	1,000		1,000	
Insatisfeito	19,6 (16,7–22,8)	3,081 (2,228–4,258)	< 0,001	3,132 (2,266–4,329)	< 0,001

OR: *odds ratio*; OR_a_: *odds ratio* ajustada; IC95%: intervalo de confiança de 95%; APS: atenção primária à saúde; HAS: hipertensão arterial sistêmica.

^a^ Ajustado por região, sexo, escolaridade, polifarmácia, ajuda, emergência e internação.

^b^ Ajustado por sexo, escolaridade, idade, ajuda, emergência e internação.

^c^ Ajustado por sexo, escolaridade, idade, emergência e internação.

## DISCUSSÃO

Os resultados demonstraram que a não adesão, seja ela por dificuldades de acesso na última vez, por não uso na semana anterior ou declarada, é um evento mais prevalente entre os insatisfeitos com os serviços em todas as dimensões estudadas e está associada à insatisfação com as dimensões oportunidade e adequação.

A insatisfação com a dimensão DISP, que avaliou as barreiras enfrentadas pelos usuários para conseguir o medicamento no serviço de saúde e para usá-lo em casa, não se mostrou associada à não adesão. Porém, ao se observar os valores da NAF, que são três vezes maiores que as outras medidas (NA7 e NAD), a falta do medicamento aparecer como a principal justificativa para a NA7 e as prevalências de não adesão na dimensão DISP serem maiores entre os insatisfeitos, fica visível a dificuldade de acesso a medicamentos na APS no Brasil, fator determinante para o não uso dos medicamentos e a insatisfação dos usuários^25,26^.

O acesso gratuito via SUS atende a importante parcela da população e veio melhorando com os anos, porém ainda permanecem dificuldades^25^. A dificuldade para conseguir os medicamentos de maneira regular e gratuita esteve associada à não adesão em outros estudos no Brasil^25^.

A Pesquisa Nacional de Saúde de 2019 demonstrou que a obtenção de todos os medicamentos receitados, por via pública e/ou privada, é uma realidade para 85% dos brasileiros. Pela via pública, exclusivamente, a proporção de pessoas que conseguiram pelo menos um medicamento receitado foi de 30,5%, demonstrando que há obstáculos para o acesso no âmbito público^30^. A dificuldade de acesso público é confirmada pelos resultados da avaliação Contas SHA para o Brasil (contas de saúde na perspectiva da contabilidade internacional), que demonstrou que, de 2015 a 2019, cerca de 90% dos gastos com medicamentos e artigos médicos foram realizados direto do bolso das famílias^31^.

A insatisfação com a dimensão OP/CO, associada a NA7 e o esquecimento, foi a principal justificativa apresentada pelos entrevistados, excluída a falta do medicamento. Essa associação, ou seja, insatisfação com a existência dos serviços no lugar e quando o usuário precisa e o esquecimento, pode estar relacionada à construção de crenças negativas sobre os serviços de saúde, incluindo as experiências vividas na busca pelos medicamentos nos serviços de dispensação^32,33^. As preocupações com o tratamento são muitas vezes “mascaradas” por trás do “esquecimento”. Estudos mais aprofundados demonstram que as crenças negativas sobre os medicamentos e os serviços de saúde funcionam como promotoras do esquecimento^32,33^.

Outro fator importante é que o esquecimento, caracterizado como não adesão não intencional, parece não ser tão inconsciente ou aleatório assim. O esquecimento pode funcionar como um primeiro micro experimento para a futura não adesão intencional, quando o paciente decide ativamente renunciar à terapia prescrita^2,34^.

A insatisfação com a dimensão AD esteve associada à NAD, com a não adesão entre os insatisfeitos sendo três vezes a dos satisfeitos. Tal fato pode ser explicado porque os pacientes que declaram abertamente que não seguem as prescrições de medicamentos são caracterizados como não aderentes intencionais, que são significativamente mais propensos a duvidar de sua necessidade pessoal dos medicamentos, a ter maiores preocupações em tomá-los, além de serem mais críticos diante das diferenças entre expectativa e realidade dos serviços^2,34^. Uma possível relação inversa entre NAD e AD não pode ser descartada com dados transversais, pois fator e desfecho foram coletados ao mesmo tempo, mas outros estudos colocam a insatisfação como preditora da não adesão^35,36^.

Estudo de Barreto et.al.^17^, com pacientes atendidos em 90 serviços de APS (unidades básicas de saúde e unidades de saúde da família) e em uso de anti-hipertensivos, observou associação entre insatisfação com os serviços APS e a não adesão ao tratamento para hipertensão. Os serviços que mais causavam insatisfação eram o atendimento da recepção (atendente) e o agendamento de consultas médicas. Na Síria^37^ a não adesão aos medicamentos anti-hipertensivos esteve associada a insatisfação com os serviços de saúde, no tempo de consulta, na explicação do médico sobre a doença e na falta de medicamentos (gratuitos) nas unidades dispensadoras. Lihara et al.^34^ encontraram associação entre insatisfação com a equipe de saúde, principalmente relação interpessoal, e a não adesão ao uso de medicamentos para DCNT no Japão.

Na literatura, a influência da satisfação com os serviços de saúde na adesão ao uso de medicamentos é explicada pelos modelos da autorregulação em saúde^35^ e crenças em saúde, que têm por objetivo explicar e predizer comportamentos em saúde, e que apresentam semelhanças entre si^38^. O modelo crenças em saúde faz isso por meio de seis dimensões da percepção humana: susceptibilidade percebida, severidade percebida, benefícios percebidos, barreiras percebidas, autorregulação e estímulos para ação. Nelas, fatores como crenças, experiência com serviços de saúde, relacionados à doença, aos tratamentos e aos pacientes influenciam tanto a satisfação como a adesão^38^_._

A adesão é um comportamento em saúde mais complexo que julgar-se satisfeito e envolve fatores preditores diretos e mediadores, e a satisfação já foi observada como ambos. O que se sabe é que a satisfação é componente para adesão, quanto mais insatisfeito menos aderente, mas ainda não se tem clareza se a satisfação não é uma característica casual dos aderentes^35,38^.

Neste trabalho, a insatisfação foi construída por meio da avaliação do acesso a produtos e serviços pelos usuários de medicamentos da APS. O acúmulo de barreiras ao acesso construiu a insatisfação e esta esteve associada a não adesão. Tal resultado pode ser explicado pela dimensão barreiras percebidas do modelo crenças em saúde, no qual mais barreiras indicam maior insatisfação e menor adesão^39^. As dimensões barreiras percebidas e benefícios percebidos se mostraram consistentemente os mais fortes preditores para comportamentos em saúde^36^.

A dimensão AD apresentou as menores taxas de insatisfação do estudo. Tal resultado pode demonstrar que há adequação dos serviços e produtos às expectativas dos usuários e estes se sentem satisfeitos. Porém, é preciso considerar alguns fatores que influenciam o padrão majoritariamente positivo das avaliações de satisfação, como usuários serem pouco críticos em relação a qualidade dos serviços recebidos, seja por desinteresse, por falta de modelos de comparação ou de visão de si mesmo como ator do processo e pelo viés de gratidão (*gratitude bias)*, mais presente em serviços de saúde públicos e populações periféricas^5^.

Ao observar as variáveis das dimensões DISP e AD, que apresentaram maiores taxas de insatisfação, com exceção das relacionadas à falta acesso (já discutido anteriormente), destacam-se os problemas no uso domiciliar (dificuldade de ler a embalagem e de lembrar de tomar) e o momento da dispensação (insatisfação com a privacidade no atendimento, com o recebimento das informações sobre o uso, como guardar os medicamentos em casa e com o conforto do ambiente).

Esses resultados são um alerta sobre em quais situações residem as dificuldades e insatisfações dos pacientes, e suas soluções envolvem SF centrados no paciente, como dispensação com qualidade e informações por profissional capacitado em ambiente apropriado para cuidados em saúde, acompanhamento clínico do uso de medicamentos, treinamento de habilidades para autocuidado em DCNT, ainda em construção no Brasil^40^.

Avaliações recentes sobre a implantação, fidelidade e desempenho dos SF no SUS apresentam um quadro de grandes diferenças regionais, atividades voltadas principalmente para logística do produto, ainda com problemas de abastecimento, estrutura, insuficiência de farmacêuticos na dispensação e acompanhamento de tratamentos e que deixa o uso do medicamento sob responsabilidade dos pacientes^40^. Essa organização desfavorece a corresponsabilidade entre profissionais/serviços e pacientes no manejo de doenças, na monitorização de pacientes em uso de medicamentos, na adesão aos tratamentos e na obtenção de melhores resultados em saúde.

A medida da insatisfação com uso da TRI em perguntas fechadas apresentou limitação por deixar de contemplar crenças, modos de vida, concepções sobre saúde-doença e expectativas dos usuários de maneira direta, mas tem como fator positivo a diminuição do viés de gratidão e outros fatores sociais que influenciam as pesquisas diretas sobre satisfação^12^. Outras limitações do estudo são a característica transversal do banco de dados, o não uso de todas as variáveis de ajuste para insatisfação e adesão descritas na literatura devido a indisponibilidade no banco de dados, a dificuldade de acesso aos dados que deveriam ser públicos, o uso do autorrelato e medidas pontuais de não adesão, a temporalidade dos dados (2013–2014) e o efeito da falta de medicamentos nos desfechos NAF e NA7.

A limitações foram atenuadas pela utilização de período recordatório curto (sete dias) para um dos desfechos, pela relação entre fator e desfecho não ser tempo dependente, pela relevância dos dados PNAUM, maior *survey* sobre uso de medicamentos do Brasil, e ainda único em sua relevância, pela falta de pesquisas no Brasil que avaliem a associação entre insatisfação com serviços de saúde e a não adesão ao uso de medicamentos por meio de modelos teórico e metodológico construídos para esse fim e pela heterogeneidade das características dos SF da APS SUS, que faz com que muitos municípios brasileiros vivam hoje a realidade PNAUM de 2014. Além disso, a organização do modelo de Esher^11^ ajudou a visualizar a diversidade de aspectos intermediários que permeiam a relação entre usuário e serviços de saúde, possibilitando investigar como os usuários possuem diferentes ligações nas unidades de dispensação e as dimensões que influenciam a satisfação/Insatisfação.

## CONSIDERAÇÕES FINAIS

A maioria dos usuários APS estava satisfeita com os SF, porém os insatisfeitos foram mais não aderentes ao uso de medicamentos e a insatisfação com a existência dos serviços no local e tempo requeridos pela saúde dos usuários (oportunidade), e com a diferença entre os produtos/serviços oferecidos e as expectativas e necessidades dos usuários (adequação), esteve associada à não adesão ao uso de medicamentos.

São necessários mais estudos sobre a adesão aos medicamentos em pacientes de DCNT no Brasil, e principalmente no SUS, com coleta de dados longitudinais e análises mais profundas das causas. Para enfrentar o problema é preciso conhecê-lo e assim propor caminhos segundo as causas mais importantes e a realidade dos serviços de saúde brasileiros.

Para mitigar uma insatisfação que influencia a não adesão e que se baseia na dificuldade de se obter os medicamentos, na falta de serviços que atendam as expectativas e necessidades dos usuários em locais e tempo oportunos, o Brasil precisa reforçar as políticas públicas de acesso aos medicamentos, implementar e promover SF centrados nos pacientes, considerando suas necessidades e perspectivas sobre os serviços recebidos com atividades de gestão, dispensação e acompanhamento clínico realizadas por farmacêuticos. Iniciativas recentes como a (re)implantação das equipes e-multi com carga horária para farmacêuticos^45^ e a publicação das Diretrizes Nacionais para o Cuidado Farmacêutico no âmbito do SUS^10^ demonstram passos na construção dos SF na busca de resultados positivos em saúde.

## Data Availability

Os dados estão disponíveis mediante solicitação ao autor correspondente.
